# Identification of metabolite and protein explanatory variables governing microbiome establishment and re-establishment within a cellulose-degrading anaerobic bioreactor

**DOI:** 10.1371/journal.pone.0204831

**Published:** 2018-10-05

**Authors:** Stephen J. Callister, Lee Ann McCue, Amy A. Boaro, Brian LaMarche, Richard A. White, Joseph M. Brown, Birgitte K. Ahring

**Affiliations:** 1 Biological Sciences Division, Pacific Northwest National Laboratory, Richland, Washington, United States of America; 2 Department of Biological Systems Engineering, Washington State University, Pullman, Washington, United States of America; 3 Environmental Molecular Sciences Division, Pacific Northwest National Laboratory, Richland, Washington, United States of America; 4 Bioproducts, Sciences and Engineering Laboratory, Washington State University Tri-Cities, Richland, Washington, United States of America; 5 Gene and Linda Voiland School of Chemical Engineering and Bioengineering, Washington State University, Pullman, Washington, United States of America; The University of Akron, UNITED STATES

## Abstract

Proteins, metabolites, and 16S rRNA measurements were used to examine the community structure and functional relationships within a cellulose degrading anaerobic bioreactor. The bioreactor was seeded with bovine rumen fluid and operated with a 4 day hydraulic retention time on cellulose (avicel) as sole carbon and energy source. The reactor performance and microbial community structure was monitored during the establishment of the cellulose-degrading community. After stable operation was established in the bioreactor, the mixing intensity was increased in order to investigate the effect of a physical disruption of the microbial community structure. Finally, the original conditions were re-established to understand the stability of the microbial community after a perturbation. All factors measured were found to be inter-correlated during these three distinct phases of operation (establishment, perturbation and re-establishment). In particular, the return of community structure and function to pre-perturbed conditions suggests that propionate fermentation and acetate utilization were the explanatory factors for community establishment and re-establishment.

## Introduction

The successful operation of a bioreactor employing a microbial community for industrial and biotechnological applications is often based upon the optimization of operational parameters and environmental factors that have been empirically investigated and modeled to provide process performance and stability. The established microbial community driving the biological processes within the bioreactor is typically a consortium acquired from an external environment, such as a bioreactor already in operation, or from a natural environment. The introduction of the inoculum microbiome to the bioreactor represents an important step in the start-up of the bioreactor, and it is generally accepted that the development of community networks within a new environment represents a critical factor for achieving the desired process performance through preservation of diversity and functional redundancy [1–3]. In many instances, the established community only partially resembles the initial consortium as the final populations thriving in the bioreactor must acclimate to controlled conditions within the bioreactor.

The anaerobic methane generating bioreactor represents a model system for investigating community establishment and function; where, the metabolic network proceeding from hydrolysis to methanogenesis is driven by interdependent interactions that must develop following introduction of the consortium to a new environment. Then, this interdependence must persist in order for methanogenesis to continue. Numerous studies have focused on anaerobic bioreactors, measuring and comparing differences in community structure from the point of inoculation through stable operation. Similarities and differences between the initial consortium and the established microbiome are often ascribed to environmental factors, or to variations in bioreactor operation or bioreactor design (i.e. F/M ratio–food to microorganism ratio, dilution rate, temperature). For example, Vanwonterghem et al., 2014, discussed the synchronicity of replicate digesters, noting that during bioreactor start-up the many hydrogenotrophic methanogens within the inoculum could each have come to dominance independently if a major tenet of neutral theory, which ignores the innate differences between species [4], represented a major driver in community establishment and dynamics [5].

Evaluating the influence of such environmental factors on community dynamics can be achieved through investigation of community establishment and re-establishment as a consequence of an environmental pulse perturbation. For example, we recently investigated pre- and post-community and functional responses to a pH pulse perturbation applied to an anaerobic cellulose degrading bioreactor [6]. Multi-omics measurements made before and after the perturbation led to our finding that a drop in pH (6.8 to 5.5) followed by its return played a role in altering community structure, but did not significantly alter functions associated with the microbiome, suggesting the presence of functional redundancy within the microbiome.

While the above study demonstrated a highly conserved proteome before and after the perturbation, our focus here was on the identification of variables, measured using multi-omics capabilities that explain community and functional establishment during bioreactor start-up and re-establishment that followed after a pulse perturbation brought the microbiome to the brink of collapse. All multi-omics measurements were made on samples acquired from the bioreactor over a span of 120 days of operation. To evaluate the functional proteomics response we used substantial metagenomics sequencing on samples collected and pooled during the span of the bioreactor operation.

We perturbed the established community by increasing the mixing intensity within the reactor vessel. Selection of the perturbation was based on previous research that suggests that different mixing intensity impacts process performance [7–10] (particularly during bioreactor start-up [11]) through physical disruption of interdependent interactions within the community. From these findings, we hypothesized that the community, metabolite, and protein functional patterns before, during, and after the perturbation would reveal metabolic activities and interactions of the members during community establishment and re-establishment. We observed that global patterns between VFA metabolites, population abundances, and proteomes are highly correlated before, during, and after the mixing perturbation. Here, we observed propionate as the key explanatory variable for community and functional re-establishment, while acetate and caproic acid formation/utilization were also important variables during community establishment.

## Materials and methods

### Bioreactor operation

The laboratory scale anaerobic continuous flow bioreactor used for this study was inoculated with bovinerumen fluid acquired on-site from a mobile slaughter operation. The bioreactor consisted of a 3 L sealed glass vessel, water jacket, temperature probe, pH probe, sampling ports, and mixer system (Z611000320, Applikon fermentor, Schiedam, Netherlands). The reactor media consisted of BA media [12] supplemented with 0.1 g/L yeast extract, 0.1 g/L tryptone, and with 10% (by volume) rumen fluid clarified by centrifugation at 3000 x g for 30 minutes at 4°C. Substrate was 10 g/L Avicel microcrystalline cellulose (Sigma Aldrich). The media was sterilized by autoclave using the liquids setting at 121°C for 30 minutes (MLS-3781L, Sanyo, Panasonic Healthcare Company of North America, Wood Dale IL). Detailed operation of this bioreactor has been described elsewhere [6]. In summary, the reactor contents were mixed at 200 rpm, temperature was maintained at 39 ± 0.5°C by water recirculation through the water jacket, and pH maintained at 6.8 ± 0.2 through real-time alkalinity adjustment by addition of 1N NaOH. Effluent was passively pumped from the reactor via displacement by fresh media (solids and hydraulic retention time of 4-days). Anaerobic conditions were maintained through microbial gas production and the addition of media purged with a 20:80 CO_2_:N_2_ gas mix (Oxarc, Spokane WA).

### Bioreactor monitoring

Fixed solids, volatile solids, headspace gas content, and volatile fatty acids (VFAs) were measured using standard protocols [6, 13]. In brief, 10 VFAs: formate, acetate, propionate, butyrate, isobutyrate, valerate, isovalerate, caproic acid, isocaproic acid, and heptanoic acid were measured using a Dionex Ultimate 3000 HPLC (Sunnyvale, CA) equipped with autosampler, Shodex RI-101 detector (Tokyo, Japan), and 300 x 7.8 mm Aminex HPX-87H column with a 30 x 7.8 mm Cation H guard column (Bio-Rad, Hercules CA). The HPLC mobile phase consisted of 0.005 M H_2_SO_4_ maintained at 60°C at a flow rate of 0.6 ml/min. Pre-mixed standards for VFA analysis were purchased from Sigma-Aldrich (St. Louis, MO) and ranged from 1 mM to 10 mM for valeric, isovaleric, caproic, isocaproic, formic, and heptanoic acids, and from 1 mM to 25 mM for acetic, propionic, butyric and isobutyric acids. Headspace gas concentrations of CO_2_, H_2_, CH_4_, and O_2_ were determined by removing a set volume (5 ml initially, then subsequently 2 ml) of gas from the headspace, injecting this into a 35 ml serum vial purged and filled with N2, then injecting an equivalent volume into a SRS 300 AMU Gas Analyzer (Vineland, NJ) according to Boaro, et al., 2014 [6]. Gas production rates were not determined beyond measured concentrations within the headspace.

### Perturbation

Approximately, 50 days following inoculation, mixing within the vessel was increased through an increase in impeller (axial flow impeller) speed from 200 revolutions per minute (rpm) to 400 rpm and maintained at this speed for 49 days. An estimate of average shear rate was calculated in the absence of baffles as 214 s^-1^ for 200 rpm and 428 s^-1^ for 400 rpm [14]. These estimates for shear rate are within the range reported by others in measuring the microbiological effects of shear within continuously stirred anaerobic bioreactors [11]. The impeller speed was reduced to 200 rpm on 99 days post inoculation.

### Sample collection

Samples for pyrosequencing and proteomics were harvested over a 120-day span beginning at the day of reactor inoculation. Samples were stored at -80°C. Samples (10–20 ml) for sequencing and proteomics were harvested and centrifuged for 10 minutes at 21°C, 10,000 rpm (10,600 x g) using an Eppendorf 5804 centrifuge (Hamburg, Germany), after which the supernatant was discarded and the pellets were flash frozen in liquid nitrogen. Samples selected for proteomics and community profiling were those harvested 2, 4, 9, 10, 31, 38, 41, 43, 52, 69, 85, 99, 104, 115, and 118 days post inoculation.

### DNA extraction and pyrosequencing

DNA was extracted from bioreactor samples using a MoBio soil DNA extraction kit, following manufacturer’s instructions (MoBio, CA, USA). The V4 region of the 16S rRNA gene was amplified and sequenced at Research and Testing Laboratory (Lubbock, TX). Forty-two purified DNA samples (triplicates of 14 time point samples, DNA was not isolated from day 104 samples) were barcoded and amplified using primers 515F (5’-GTGCCAGCMGCCGCGGTAA-3’) and 806R (5’-GGACTACVSGGGTATCTAAT-3’). These primers target both bacteria and archaea, and have been shown to amplify all bacteria with few biases [15]. Sequence data were generated from the 5’ end of the amplicon (from the barcoded 515F primer) using Roche 454 FLX Titanium reagents (Branford, CT). The sequence data were processed and operational taxonomic units (OTUs) identified using mothur v.1.23 [16]. Briefly, sequences with ambiguous bases or homopolymers greater than 8 bases were excluded, as were sequences that did not align to the V4 region of the Silva 16S rDNA reference alignment [17], or that were identified as chimeric by both UCHIME [18] and ChimeraSlayer [19]. The remaining sequences were aligned to the Silva 16S rDNA reference alignment, assigned to OTUs at ≥ 97% identity (with furthest neighbor linkage), and taxonomy assigned using the RDP reference taxonomy [20]. After processing the data for quality, the triplicate datasets were compared for consistency in community membership (the observation of an OTU within all three replicates) and structure; one replicate from day 2 and one replicate from day 9 were in disagreement and eliminated. The remaining replicates from each of the 14 samples were then combined by calculating the median sequence counts for each time point, and the median sequence counts observed in each OTU for each time point. The sequence counts per sample were then normalized [21] by extracting a random subsample of 2500 sequence counts (and their assignment to OTUs) from each time point sample for further analyses.

### Metagenomic library preparation

DNA extracted from a selected number of bioreactor samples were pooled based on total DNA mass (~5 μg) and periods of bioreactor operation encompassing 2, 4, 9, 10 days post inoculation (Library 1); 38, 41, and 43 days post inoculation (Library 2); 69, 85, and 99 days post inoculation (Library 3); and 115, 118, 140 days post inoculation (Library 4). Illumina library construction was completed as previously described [22]. In short, DNA was sheared by ultrasonication (Covaris M220 series, Woburn, MA, USA), A-tailed, ligated with Illumina TruSeq adapters, then library fragments were purified and small fragments removed using AMPureXP magnetic beads (Beckman Coulter, Danvers, MA). The libraries for pools 1 to 4 had separate Illumina indexes then were combined and sequenced on an Illumina HiSeq2500 using the Rapid mode 250 bp paired-end format at WSU Spokane (Spokane, Washington, USA). Metagenomic sequence data for each library has been deposited with the Open Science Framework (DOI 10.17605/OSF.10/8WBJN).

### Metagenome analysis

Metagenomic quality control, de novo assembly, and annotation was completed as previously described [22]. In summary, raw paired-end Illumina reads (250 bp in FASTQ format) were merged at read overlaps using FLASH (https://ccb.jhu.edu/software/FLASH), after which φX174 viral genome spike-in was removed using Bowtie2 [23]. The reads were trimmed and adapters removed using Trimmomatic [24] and quality checked with FastQC (http://www.bioinformatics.babraham.ac.uk/projects/fastqc/). Cleaned and quality controlled reads were assembled with de Bruijn graph assembler MEGAHIT [25]. The resulting contigs were subsetted to contigs >1 kbp, then quality controlled and checked with custom Python scripts, which provide assembly size, the number of contigs, contig length distribution, and N50 values. For quantification, reads were mapped back to quality-controlled contigs using Bowtie2 and annotation was completed with Metapathways2 [26]. Assembled metagenomic contigs were binned using MaxBin2 [27]. The resultant genomic bins were characterized using CheckM [28] for taxonomic assignment, completeness, and contamination metrics.

### Protein extraction

Proteins were extracted from cell pellets harvested in conjunction to samples harvested for community profiling. To each sample, a lysis solution was added consisting of 2% SDS and 1% DTT in 100 mM ammonium bicarbonate. Samples were vortexed then transferred to pulse tubes (no lysis disk) and subjected to 10 cycles of pressure (20 seconds at 35,000 psi), allowing the sample to return to ambient pressure between each cycle, using a barocylcer (Pressure BioSciences Inc., South Easton, MA). Following lysis, cell debris was collected by centrifuging at 4°C for 15 min at 14,000 rpm (~13,000 x g). The supernatant fraction was transferred to a 2.0 ml tube (siliconized), and fresh lysis buffer added to the cell debris, followed by an additional 20 cycles of pressure as described above. From the combined supernatant fractions, 1 ml was added to a cold (kept on ice) solution containing 50% methanol, 12.5% chloroform, and 37.5% nanopure water, vigorously mixed, allowed to stand for 2 min at -20°C, then centrifuged at 4°C for 5 minutes at 12,000 rpm (~10,000 x g). The upper layer (containing protein) was removed and 3 ml cold methanol added, followed by additional mixing and centrifuging as described above. The protein pellet was allowed to dry at room temperature, dissolved in denaturing and reducing solution containing 100 mM, 8 M urea, and 10 mM DTT. After an incubation period of 1 hr at 60°C, the solution was diluted, and digested using Trypsin as described previously [29]. Following digestion, sample clean-up was performed using solid phase extraction strong cation exchange (SPE SCX) and peptide concentration measured as described previously [29].

### Proteomics data generation

Peptide samples were analyzed in triplicate using an LTQ Orbitrap Velos mass spectrometer (Thermo Scientific, San Jose, CA) coupled to a reversed-phase HPLC separation using in-house manufactured columns (60 cm x 360 μm o.d. 75 μm i.d. fused silica capillary tubing) packed with 3 μm Jupiter C18 stationary phase (Phenomenex, Torrence, CA). A detailed description of HPLC operating conditions including mobile phase composition is described elsewhere [30]. In brief, 5 μl of peptides (0.7 μg/μl) were injected to the HPLC and subject to an exponential gradient of acetonitrile [30]. A 40-cm length of 360 µm o.d. x 15 µm i.d. fused silica tubing was used to split ~20 μl/min of flow before it reached the injection valve (sample loop), which controlled the gradient speed under conditions of constant flow (~400 nl/min).

Separated peptides were ionized (positive) using an electrospray ionization interface (manufactured in-house; no sheath gas or make-up liquid was used) that consisted of chemically etched electrospray emitters [31] (150 mm o.d. 20 mm i.d). The mass spectrometer was operated using a heated capillary temperature and spray voltage of 300°C and 2.2 kV, respectively. Data was acquired for 100 min beginning ~60 min after sample injection. High-resolution parent ion mass spectra (AGC 1x10^6^) were collected from 400–2000 m/z at a resolution of 60k followed by generation of data dependent collision induced dissociation (CID) MS/MS high-resolution spectra, centroid mode, at a resolution of 7500 (collision energy 35%, activation time 30 ms, (AGC 5x10^4^) of the 10 most abundant ions using an isolation width of 3 Da. Charge state screening was not enabled. A dynamic exclusion time of 60 sec was used to discriminate against previously analyzed ions.

### Proteomics data analysis

Fragmentation ion spectra (tandem mass spectra) generated from LC-MS features were searched using MS-GFDB [32] to assign peptide sequences using a parameter file for high-resolution CID spectra allowing for: 1) methionine oxidation M (+15.9949), 2) proline oxidation P (+15.9949), 3) a parent ion tolerance of 20 ppm, and 4) partial tryptic search. For generation of theoretical spectra, the assembled and annotated metagenomics databases described above were utilized (Metagenomics Analysis). Only peptides at least 6 amino acid residues in length having a spectral probability of less than 1x10^-9^ were retained. MS-GFDB computes the probability (p-value) that a peptide spectrum match is false. A protein was considered present within a bioreactor sample if it was identified by greater than 2 unique peptides. Protein abundances were estimated by summation of spectral counts generated from the identifying peptides. KEGG Orthology (KO) identifiers were assigned as described previously [22], and metabolic pathways reconstructed for observed proteins. For quantification of pathways, abundances of proteins identifying a pathway were summed and normalized by the protein number. Raw tandem mass spectra files and 16S rDNA pyrotag data have been deposited at PeptideAtlas (http://www.peptideatlas.org; Accession PASS00620).

### Data integration

Global OTU, VFA, and proteomics data (protein identifications) were analyzed using tools available in PC-ORD 6 (mjm software designs, Gleneden Beach, OR). Bray-Curtis similarity (inverse of dissimilarity scores) was used to construct a matrix for each data type and similarity between data types was evaluated using the Mantel test (999 permutations; p-value <0.01). Sampling events consisting of OTUs were visualized using Nonmetric Multidimensional Scaling (NMDS). The number of dimensions to include in the ordination was determined using PC-ORD’s stress test algorithm (Kruskal’s stress formula 1) with results compared to Monte Carlo test results (300 runs). Dimensions greater than 2 showed no decrease in stress and were different from stress results obtained from the Monte Carlo analysis (p-value < 0.01). Correlations between the response matrix (OTUs), and explanatory matrices of variables (inverse of Bray-Curtis values from VFAs abundance, inverse of Bray-Curtis from spectral counts of proteins and pathways) were displayed using joint plots, where the vector represents the hypotenuse of the correlations (Kendall’s tau) between the axes of the response matrix [33].

## Results

### Bioreactor monitoring

**Fig 1:**
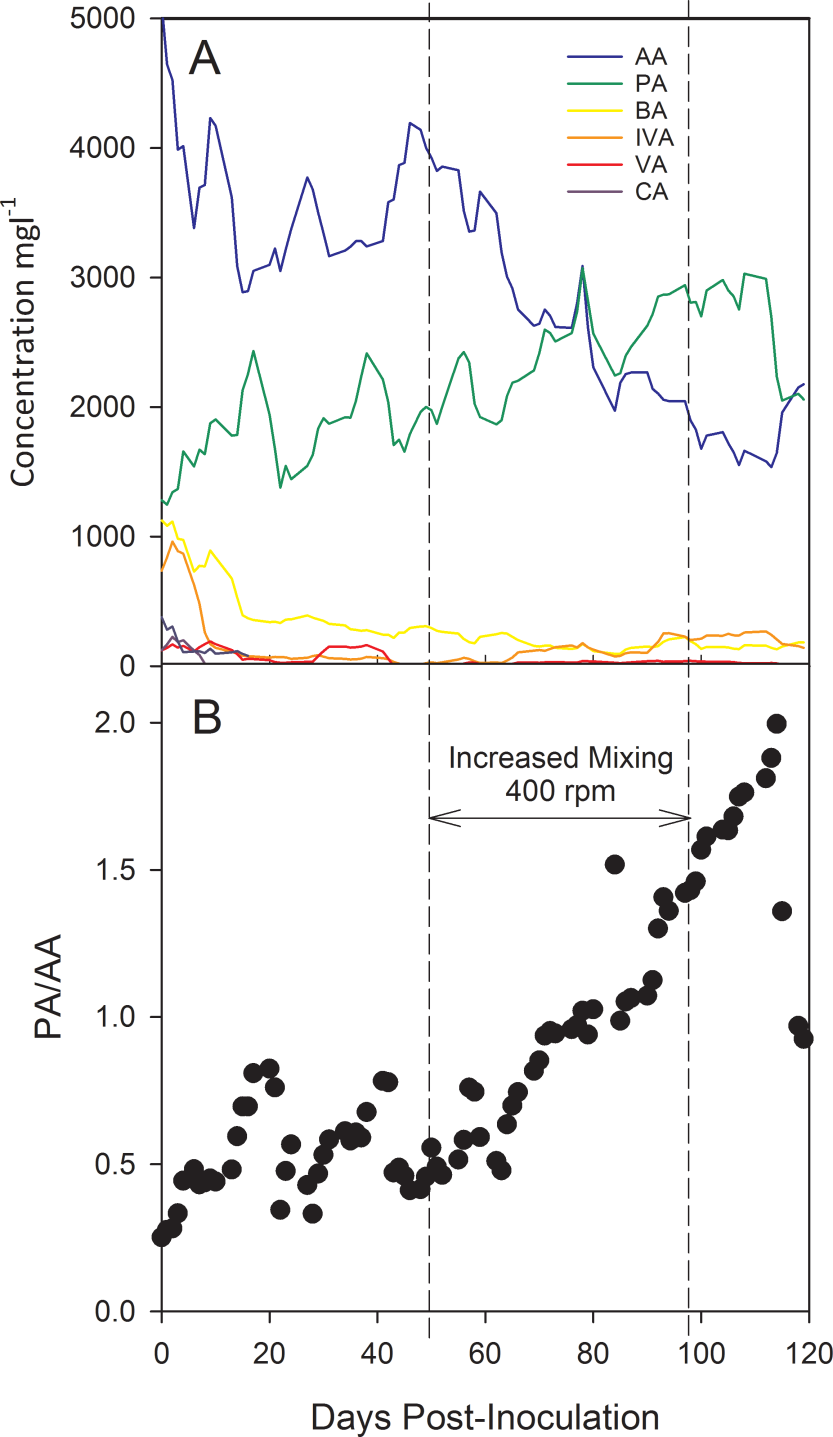
Bioreactor performance. (A) Trend in VFA metabolites over the course of bioreactor operation. AA, acetate; PA, propionate; BA, butyrate; IVA, isovalerate; VA, valerate; CA, caproic acid. (B) Ratio of propionate to acetate as an indicator of bioreactor process performance. Ratios above 1.4 are considered an indication of imminent process failure. The ratio of PA to AA was observed to increase substantially after the impeller speed was doubled (200 rpm to 400 rpm; dotted vertical lines) shortly after 50 days of bioreactor operation. A return of the impeller to 200 rpm at 99 days of operation resulted in the drop of the ratio.

Measurement of 6 VFA metabolites (Fig 1A), including acetate, propionate, butyrate, isovaleric acid, valeric acid, and caproic acid were measured over the course of the bioreactor operation. Concentrations of formate and isocaproic acid were measured in only a single sampling event, and isobutyrate and heptanoic acid were not detected in any of the samples. A carbon mass balance based upon concentrations (measured 40 days post-inoculation) of cellulose, acetate, propionate, butyrate, isovaleric acid, and gas (CO_2_ and CH_4_) was in 86% agreement with measured concentrations (S1 Table). Acetate had the highest initial concentration until approximately 60 days post-inoculation when it was superseded by propionate that continued to accumulate in the bioreactor until approximately day 105. The early accumulation of valeric, isovaleric, and caproic acids observed within the first 4-days of inoculation are indicators of early fermenter stress, as these VFAs are often measured in relatively low concentrations within stable bioreactors. Evidence suggests that accumulation of butyrate and isobutyrate are also indicators of short term stress [34, 35]; however, butyrate did not show the same rapid increase followed by a decline as observed for valeric, isovaleric, and caproic acids.

Just after 50 days post-inoculation the propionate to acetate ratio (Fig 1B) steadily climbed to above 1.4, suggesting unfavorable conditions for propionate fermentation and imminent process failure [34–36]. This increase was observed shortly after doubling the impeller speed (200 rpm to 400 rpm), indicating the disruption of syntrophic interactions, supported by a measured decrease in CH_4_ partial pressure and a rise in H_2_ partial pressure (S1 Fig). Propionate fermentation is inhibited by increased hydrogen and the accumulation of butyrate and acetate [37–39]. A return of the propionate to acetate ratio below 1.4 followed after a decrease in the impeller speed at day 105.

### Community composition

16S rRNA gene sequencing of 14 sampling events spanning 120 days of bioreactor operation yielded 1215 operational taxonomic units (OTUs), most of these (1070 of 1215) classified to 13 bacterial phyla (S2 Table). Dominant phyla included Bacteroides, Firmicutes, and Proteobacteria, each with greater than 200 OTUs. Additional phyla included Fibrobacteres, Spirochaetes, Synergistetes, Verrucomicrobia, and Actinobacteria; each with greater than 30 OTUs. Three-hundred twenty eight OTUs were assigned to 62 specific genera (S2 Table) including the cellulosic degrading genera of *Fibrobacter* (22 OTUs) and *Rumminococcus* (11 OTUs), saccharolytic genera including *Prevotella* (48 OTUs), *Treponema* (42 OTUs), and *Barnesiella* (15 OTUs), and asaccharolytic genera including *Succinoclasticum* (5 OTUs), *Sporobacter* (17 OTUs) and *Synergistes* (4 OTUs). *Acinetobacter* (37 OTUs) generally comprises several aerobic species. However, a novel *Acinetobacter* was recently isolated and sequenced from bovine rumen fluid [40], confirming pyrosequencing observations for this genus made previously on the rumen [41].

**Fig 2:**
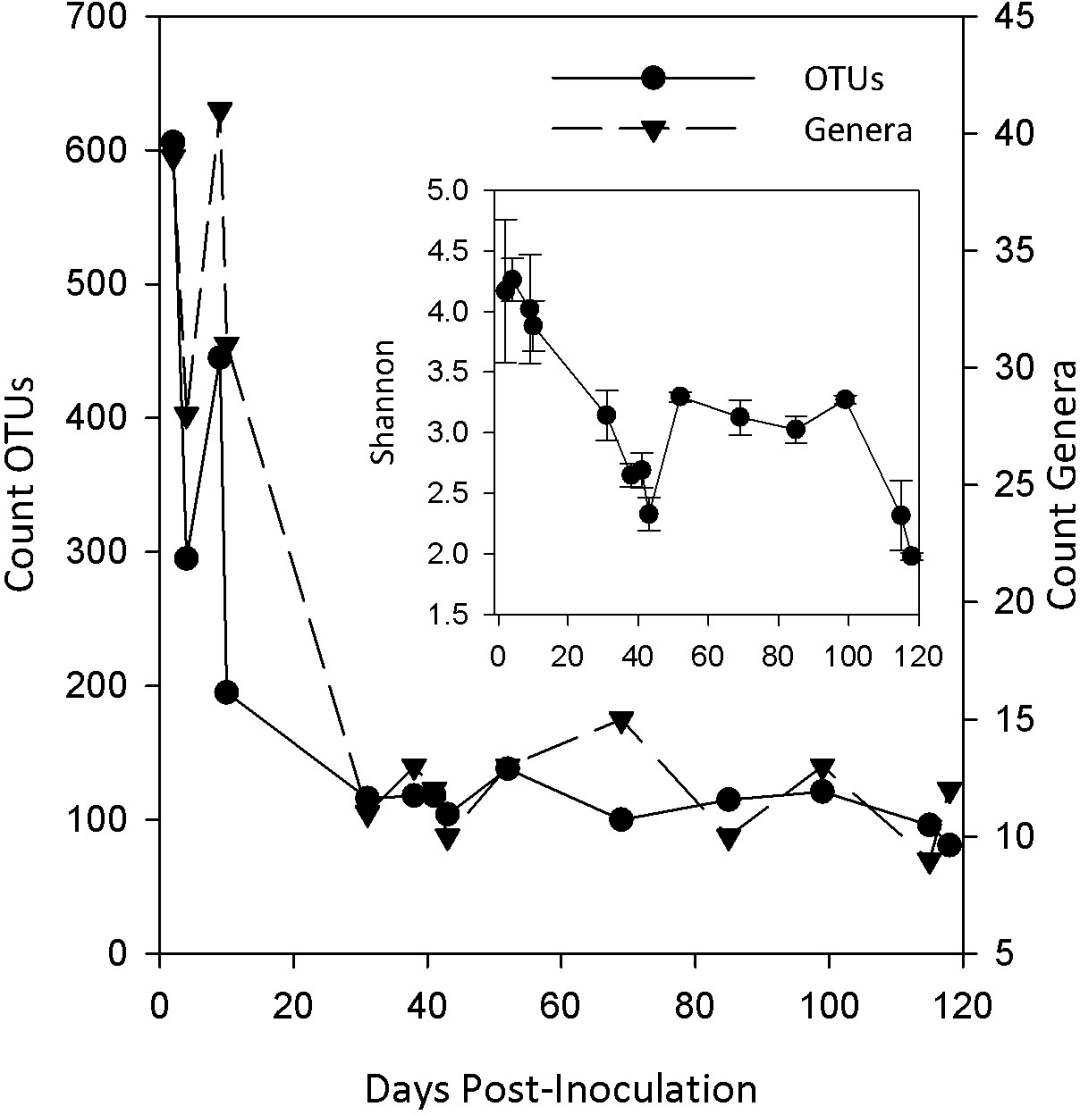
Change in OTUs and identified genera over the course of bioreactor monitoring. A steep decline was observed for both OTUs and genera during the early stage of bioreactor operation. (Inset) Change in alpha-diversity with time. Although the number of OTUs did not increase during the perturbation period, populations that were in lesser abundance prior to the perturbation increased in abundance, which would be captured by the use of the Shannon-Weaver index.

A decline in consortium membership occurred early after inoculation. Six-hundred and six OTUs observed 2 days post-inoculation rapidly dropped to 116 OTUs by day 30 (Fig 2), suggesting a loss of diversity and a loss in biomass as also evidenced by a drop in volatile suspended solids (S2 Fig). The number of genera also dropped from 39 to 11 during this same period of time. Examples of genera remaining includes *Succiniclasticum*, *Fibrobacter*, *Ruminococcus*, *Pyramidobacter*, *Synergistes*, *Treponema*, *Prevotella*, *Papillibacter*, *Barnesiella*, *Oscillibacter*, *and Sporobacter*. Alpha-diversity (Shannon Index) declined in a similar manner until day 40, where it increased in response to increased mixing within the bioreactor. This relative increase in diversity was maintained until restoration of the original impeller speed. The Shannon Index used as a measure of alpha-diversity has greater sensitivity to rarer (less abundant) populations than other measures of alpha-diversity, and although the number of OTUs did not increase during the perturbation period, some populations that were in lesser abundance prior to the perturbation increased in abundance.

**Fig 3:**
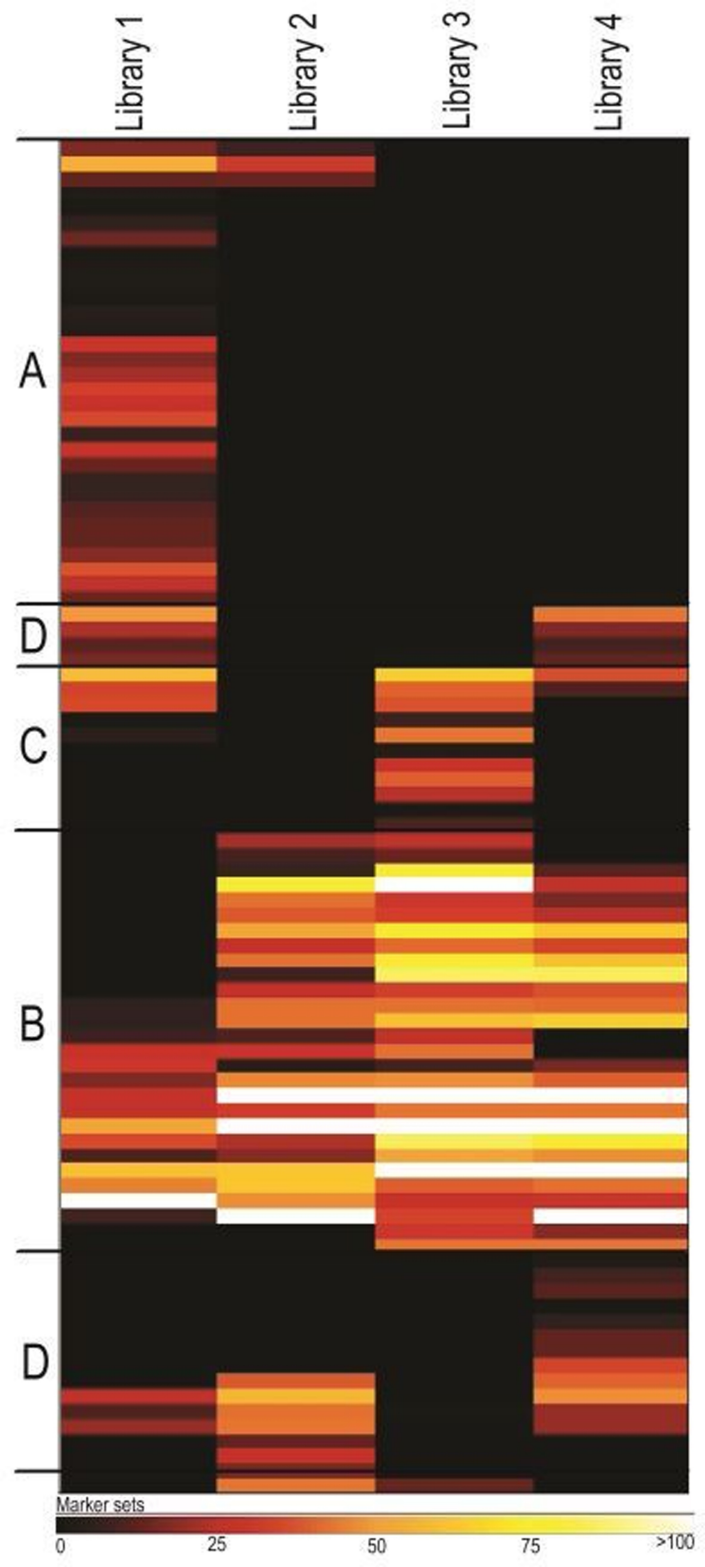
Heat map of clustered markers sets for bins assembled from each metagenomics library. Letters associated with the clusters highlight major differences between libraries suggesting community dynamics during inoculation (A), stable bioreactor operation (B), and bioreactor perturbation (C and D).

Ninety-one bins were constructed from the metagenome libraries, which were comprised of 16 phyla and 35 genera (S3 Table). Near to complete genomes (> 90% completeness) were assembled for 32 organisms, including *Fibrobacter* (100%), *Treponema* (100%), *Aminobacter* (100%), and *Pyramidobacter* (100%), and others (S3 Table). Seventeen genera identified from the 16S rRNA data were validated by the binned genomes (S2 Table), including 3 bins for *Acinetobacter* (3% to 39% genome completeness). While the 16S rRNA results were not able to confidently identify (OTUs for this domain were not observed within all sequencing replicates) the presence of archaea, 4 were binned ranging from 9% to 89% genomic completeness. The number of bins decreased between the first set of pooled samples (Metagenome Library 1) and subsequent metagenome libraries. Here, the clustering of marker sets for assembled bins (>20% completeness), revealed a large cluster (Fig 3A) composed of 25 bins having no marker sets in subsequent libraries, while 3 clusters associated with 26 bins (Fig 3B), have marker sets either within all libraries, or libraries with the exception of Library 1. Several markers sets for 26 bins (Fig 3C and 3D) appear to be directly associated with the perturbation stage of operation by: 1) their observation or lack of observation within Library 3 (pooled samples during the perturbation), or 2) their subsequent observation within Library 4 (pooled samples post-perturbation), having not been previously observed within Library 3.

### Functional composition

**Fig 4:**
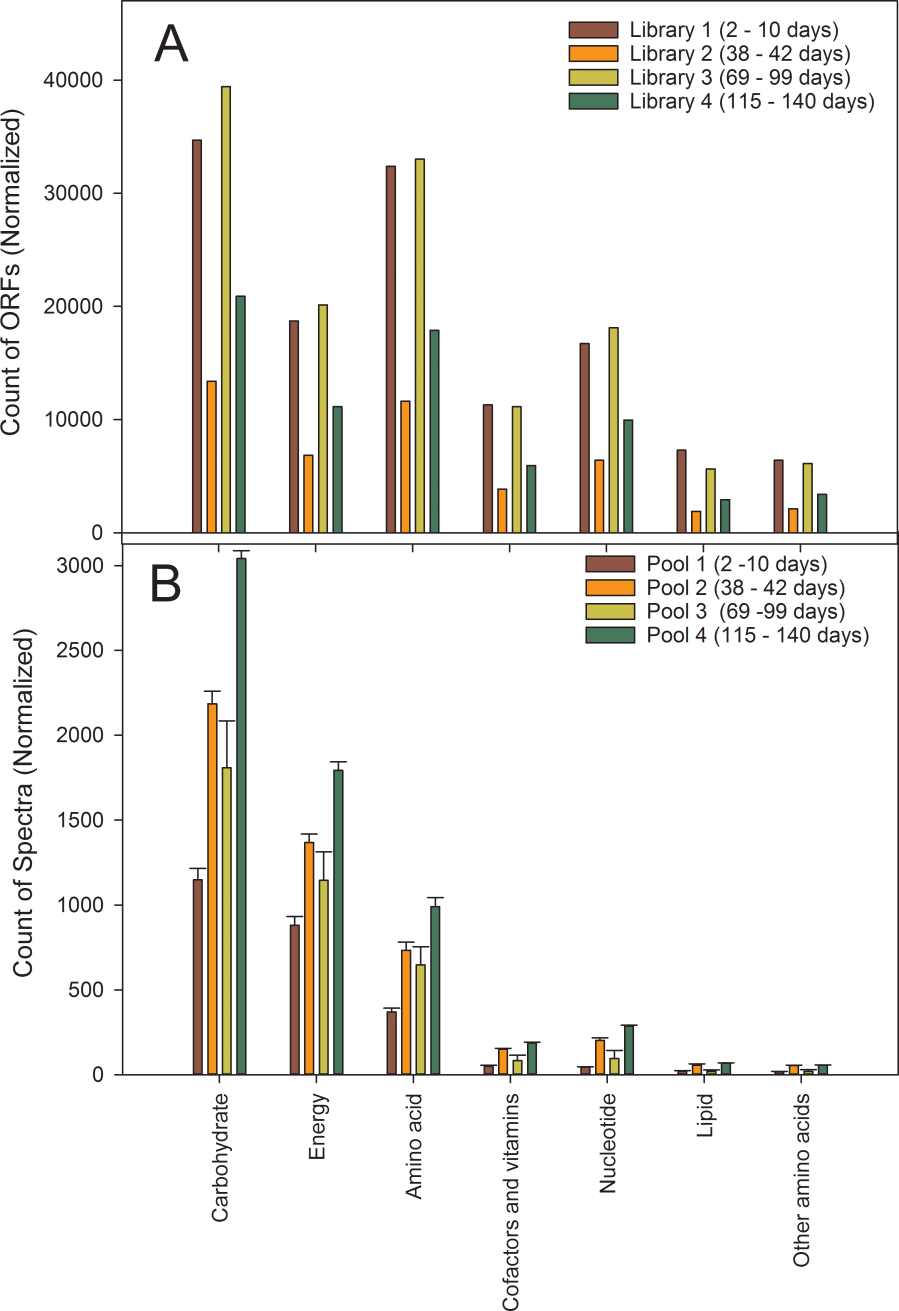
Response of major metabolic functional categories organized according to sampling events. A) metagenomics sequencing, or B) proteomics analysis. For the metagenomics libraries, the total number of ORFs was normalized by the number of sampling events pooled. For proteomics, the summed spectral counts from proteins observed from ORFs was normalized by the number of sampling events.

KEGG orthologies (KO) were assigned to 85% of the greater than 1.5 million protein-coding open-reading frames (ORFs) identified from all metagenomics libraries. Each ORF had a minimum of 60 amino acids in length. Seventy-three percent of these ORFs related to metabolism followed by genetic processing (14%). Within the category of metabolism greater than 40% of ORFs belonged to amino acid and carbohydrate metabolism (~20% in each). General energy and nucleotide metabolism represented another 20% of ORFs. Lipid metabolism and cofactor/vitamin metabolism was less than 5% of the predicted ORFs. When comparing libraries (Fig 4A), the count of ORFs for subcategories within metabolism was larger for Library 1 (2, 4, 9, 10 days post-inoculation) even after normalizing by the number of samples pooled within each library, which may have resulted from the greater diversity of organisms within the bioreactor right after inoculation. The count of ORFs (normalized) within Library 3 (perturbation) was also larger than before (Library 2) and after the perturbation (Library 4), also agreeing with the measured increase in alpha-diversity during the perturbation.

Measured peptides from high-resolution tandem mass spectra identified 7,653 proteins (S4 Table) by at least 2 or more unique peptides. A large percentage (~38%) of these observed proteins annotated as “hypothetical”,” putative”, or “unknown function” were not useful for the functional characterization of the proteome. For the rest, their placement into functional categories agreed with the metagenomes at large, namely the largest percentage of observed proteins belong to carbohydrate metabolism (42%), energy metabolism (26%), and so forth. However, when comparing functional categories across the pooled sets of proteomics samples (pooled similarly to those used for constructing the metagenome libraries) differences were observed between the number of ORFs assigned to functional categories and their actual expression. For example, proteome expression for carbohydrate metabolism, based upon summed spectral counts for annotated proteins within this category, was less for Pool 1 (analogous to Library 1) compared to all other of proteomics pools (Fig 4B). This is contrary to what was observed for annotated ORFs, where the number of ORFs was greater for Library 1 (Fig 4A and 4B). This discrepancy suggests a large proportion of the populations within the inoculum were not metabolically active. Additionally, proteome expression was highest for carbohydrate metabolism, and other metabolic categories following cessation of the perturbation (Pool 4 versus Pool 3), suggesting a functional rebound of surviving populations that were otherwise functionally inhibited during the perturbation.

### Integration of metabolite, community and functional data

**Fig 5:**
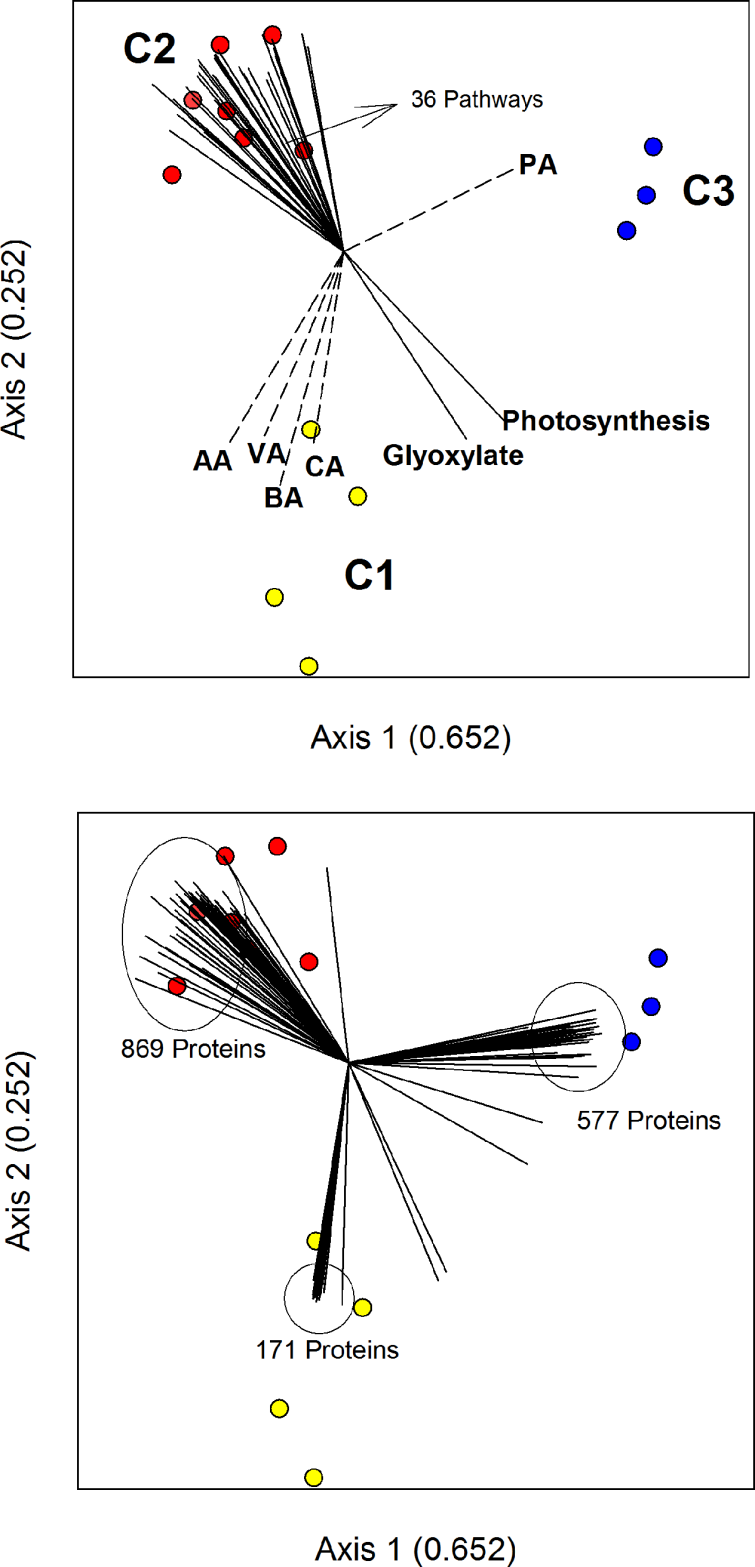
Non-metric multidimensional scaling of OTUs measured from specific sampling events. (circles) are sampling events in concert with joint plots (top) of VFA (dashed lines), functional pathways (solid lines), and proteins (bottom). Explanatory vectors for OTUs, VFAs, and proteins were filtered on their Kendall tau rank correlation with each axis; requiring -0.5 ≥ tau ≥ 0.5 to be included on the plot. Only a subset of proteins are displayed in the figure, but total number of proteins maintained after filtering is provided.

A similarity analysis (inverse of Bray-Curtis scores) performed on VFA metabolite concentrations, OTU abundances (tag counts), and peptide abundances (spectral counts) revealed a high degree of similarity in temporal patterns among the three data sets (S3 Fig), thus permitting meaningful data integration. To integrate the data sets, non-metric multidimensional scaling (NMDS) on the OTU similarity matrix grouped sampling events into three clusters (C1, C2, C3; Fig 5). Cluster C1 comprised the first 10 days of bioreactor operation post-inoculation, while C2 encompassed sampling events leading up to the perturbation (31–52 days post-inoculation) and after the perturbation (104–118 days post-inoculation). Finally, cluster C3 captured similar responses of OTUs during the period of increased mixing (69–99 days post-inoculation).

Several explanatory vectors encompassing dynamics in VFA metabolites, proteins, and pathways exhibited strong correlations by rank (Kendall’s tau, -0.50 ≤ tau ≥ 0.50) to each cluster (Fig 5). For example, propionate exhibited a stronger correlation with the community response during C3 than the other VFA metabolites, while acetate and caproic acids exhibited stronger correlations to C1. In regard to functional pathways, 38 pathways (out of 79) were retained after applying Kendall’s tau statistic, and a majority of these (36 pathways) displayed a higher degree of correlation to C1 than to the other clusters. Only two vectors describing pathways for glyoxylate metabolism and photosynthesis had higher correlations to C2 and C3, respectively. Finally, vectors representing the proteome were constructed from proteins observed within each sampling event for a given cluster (2517 out of 7653 proteins). From these 2517 proteins, 1705 passed the applied Kendall’s tau criteria (-0.50 ≤ t ≥ 0.50) for significant correlation, with the largest number (869) oriented toward C2 and the least oriented toward C1 (171). For visualization purposes, a subset of these proteins as are shown in Fig 4.

## Discussion

Each NMDS cluster pertains to different stages of community dynamics and community function during bioreactor operation, namely: 1) Early acclimation of populations (Fig 5; C1) to operational parameters and environmental conditions within the bioreactor leading to, 2) the establishment of a community (Fig 5; C2) which, 3) is temporarily modified through perturbation (Fig 5; C3), resulting in process performance decline verging upon failure. A return to community structure and function similar to the pre-perturbed state occurred following removal of the perturbation. These different stages of community dynamics and community function are generalizable to engineered bioreactors. However, because of the low retention time (4-days) the operation of a replicate bioreactor was not obtainable making the omics findings described below specific for this cellulose-degrading engineered bioreactor.

### Early acclimation of populations within the inoculum

This early period (Fig 5; C1) spanning 10 days was marked by dramatic changes in VFA concentrations and changes in the microbial consortium. The observed changes in microbial populations within the inoculum was anticipated as bioreactor environmental parameters only roughly approximated those of the rumen environment, and the bioreactor influent largely contained a single insoluble carbohydrate substrate. The decline followed by the disappearance of approximately 80% of populations (Fig 2) may have occurred from growth rates being less than the hydraulic retention time within the bioreactor (wash out), or a failed attempt at acclimation. For example, several taxa identified from OTUs and marker sets (S2 and S3 Tables) associated with explanatory vectors in C1 (Fig 5) are considered strict aerobes, or possibly facultative, including *Acinetobacter*, *Corynebacterium*, *Comamonas*, *Lysinibacillus*, and *Acidovorax*. It is possible that these genera were associated with the animal feed, or represent secondary colonization of the inoculum as the mobile slaughter house performed a bulk collection of rumen fluid. The absence of genomic bins for these genera and others (S3 Table) within metagenomics libraries subsequent to Library 1 provide additional evidence for their failure to acclimate.

Hydrolysis of insoluble cellulose is considered the rate-limiting step in carbohydrate processing within anaerobic bioreactors [42–44]. During this stage, a lack of cellulose derived carbon forced populations into a quiescent state, or forced them to scavenge endogenous carbon. Evidence for carbon scavenging was clearly observed in the proteome and metabolic pathways. Explanatory vectors (Fig 5) at the proteome level include redundancy in aconitate hydratase [EC 4.2.1.3] malate synthase [EC 2.3.3.9], malate dehydrogenase [1.1.1.37], isocitrate lyase [EC 4.1.3.1], and others (S4 Table). These enzymes connect the utilization of fatty acids for generation of endogenous carbon, and are observed as part of the persistence (quiescent) survival strategy of certain populations during hypoxia and nutrient (endogenous carbon) limitation [45–48]. Together, these enzymes make up a bifurcation of the tricarboxylic acid cycle (glyoxylate shunt) important to fatty acid and acetate metabolism. Genes for this bifurcation are largely found within aerobic, or facultative anaerobic bacteria [49].

The measurement of significant concentrations of acetic, caproic, valeric and butyric acids often distinguishes the early stage of bioreactor operation from later stages, and the multi-variate analysis performed here predicted their presence (Fig 5), as being significant to the success or failure of populations to acclimate within the bioreactor. The observation of caproate during the first 10 days indicates the occurrence of fermentative H_2_ production; an interesting process where acetogens acting on cellulose (or carbohydrates in general) initially produce an abundance of small VFAs and hydrogen (also ethanol) that provides reducing equivalents for cyclic elongation of acetate to butyrate (via acetyl-CoA) and butyrate to caproate via butyl-CoA. The subsequent decline in caproate and butyrate concentration (Fig 1A) points to the acclimation of methanogens competing for hydrogen.

### The established community

Fig 5, C2 represents a functioning acclimated bioreactor community that we maintained over a span of approximately 40 days prior to being perturbed. Ratios constructed from VFA measurements (Fig 1), increased proteome expression (Fig 4), and a preponderance of protein associated with pathways (Fig 5) pertaining to both catabolism and anabolism (i.e. peptidoglycan biosynthesis, amino acid biosynthesis, S5 Table), point to an established set of trophic interactions among community members. Observation of OTUs and marker sets from genomic bins point to 2 phenomena giving rise to these interactions. First, populations originally observed within the time span pertaining to C1 were also observed within the time span pertaining to C2 representing an initial set of interactions among dominant community members. For example, at the genus level *Fibrobacter* (S2 Table, OTU0016 and several others; S3 Table, UID2981, 100% completed genome) and *Rumminococcus* (S2 Table, OTU0009 and several others; S3 Table. UID1240, 98% completed genome) likely represent dominant cellulose degraders, whereas genomic bins associated with *Oscillibacter* (S2 Table, OTU00139; S3 Table, UID1230, 90% completed genome) and *Victivivallis* (S3 Table, UID3047, 91% complete genome) represent fermenters relying on byproducts of cellulose hydrolysis. Interestingly, bins associated with *Acidaminococcus* (S3 Table, UID1027, 93% completed genome) were assembled in both Library 1 and 2 (analogous to C1 and C2). To date, this genus has been reported to solely utilize amino acids (glutamate) for energy producing butyrate and propionate, representing an additional trophic level as part of this phenomenon [50]. It is also possible that by further analysis of the assembled genome, for this and other binned organisms, hypotheses concerning the utilization of additional substrates could be formulated. Second, OTUs and marker sets measured as part of C2, which were not originally observed as part C1 suggest further establishment or strengthening of trophic links. For example, the appearance of *Pyramidobacter* (S2 Table, OTU00167; S3 Table, UID235, 100% complete genome), *Prevotella* (S2 Table, OTU0040; S3 Table, UID2774, 76% completed genome), and *Anaerovorax* (S3 Table, UID1150, 91% completed genome).

### Disruption and re-establishment of community and functional response

The mixing perturbation imposed on the community for approximately 49-days caused significant alteration in community function (Fig 5; C3). Visible quantities of cellulose were observed within the bioreactor vessel and the number and relative abundance of glycoside hydrolase proteins dramatically decreased, indicating decreased cellulose hydrolysis. In addition, a measurable drop in methane concentration pointed to a decrease in methanogenesis, even though methanogenesis proteins were still observed during C3 (likely originating from the lone archaea binned from Library 3; ~88% completed genome), suggesting that increased shear within the bioreactor may have altered syntrophic interactions. The utilization of outer membrane associated structures provides one mechanism for syntrophic interactions between fermentative bacteria and methanogens, and within the set of explanatory vectors representing proteins directed toward C3, a large amount of redundancy was observed for flagellar associated proteins including hook (FlgE), motor (FliN/FliY), and predominantly FlaA; the latter representing one of the two major classes of outer membrane proteins making up the flagellar structure. Recent evidence done with syntrophic cocultures has pointed to the importance of flagellar, or related structures, in possible electron transfer, cell aggregation, and initiation of syntrophy [51–54]. Although not entirely clear here, the observation of these proteins and their relationship to C3 is suggestive of bacterial attempts at re-initiation of syntrophic interactions prevented by the magnitude of shear caused by the mixing perturbation, or possibly the use of these structures in strengthening cell aggregates. The observed increase in propionate during this perturbation supports the former as its bioconversion is highly unfavorable in the absence of a methanogenic partner [55].

Our observations pertaining to community establishment fit within classical ecological theory related to community development and succession (Reviewed; [56]); where, i) during community development certain populations facilitate the establishment (or inhibit the emergence) of others, while ii) populations having traits not suitable for the environment will not become established. The observed emergence of metabolic specialists (asaccharolytic) following establishment of the primary cellulose degraders demonstrated a dependence upon the functional response of existing populations through metabolic processing and exchange within a new environment. Indeed, the early observation of methane production and associated proteins, even at a relatively low hydraulic retention time (4-day) further points to the importance of these interactions in community establishment.

Global patterns between VFA metabolites, population abundances, and proteomes were highly correlated before and after the mixing perturbation that was successful at disrupting the metabolic and community network. The trend in decreasing PA/AA, and increased acetate concentrations (Fig 1) after restoration of the pre-perturbation mixing speed also suggest that the community is in early stages of recovery. Propionate fermentation appeared to be the explanatory variable contributing most to this re-establishment, while acetate and caproic acid utilization were key explanatory variables during community establishment. The reduction in propionate and re-establishment of methanogens via the observed relative abundance increase in methane generating proteins was a particularly interesting observation, as increased shear within the reactor appeared to target syntrophic interactions. The resiliency of this mutualism has been generalized by others observing disruptions in anaerobic bioreactors as an important property of these interacting populations [57].

## Supporting information

S1 Table Carbon balance based on 1 retention time (1.5L). (XLSX)

S2 Table OTUs identified from 16s rDNA reads. Taxonomy was assigned using the RDP reference taxonomy database. Values indicate the tag count for identified OTUs. Genera in bold were also identified from metagenomic binning. Sampling dates are referenced against the time of inoculation.
(XLSX)

S3 Table Bins resulting from each metagenomic library sequenced from pooled samples collected during bioreactor operation. "Maximum Completeness" refers to the most complete assembly in the case a bin was observed within more than one library. "Cluster ID" corresponds to the clusters identified in Fig 3.
(XLSX)

S4 Table Proteomes associated with pooled samples analagous to metagenomic libraries constructed from combining samples harvested over the span of bioreactor operation. Values within cells represent the summed spectral counts for peptides associated with each protein.
(XLSX)

S5 Table Pathway data associated with Fig 5. Values represent either the summed spectral count of proteins associated with a given pathway, or summed spectral counts normalized by the number of sampling events associated with a particular NMDS cluster. Cluster C2 has been parsed according to those sampling events tha occurred before the perturbation (BP) or after the perturbation (AP).
(XLSX)

S1 Fig Gas analysis results from the bioreactor headspace. CH_4_ and CO_2_ were observed during the established community; however, partial pressure for both gases dropped significantly with a rise in H_2_. These observed changes approximately coincide with an increase in impeller speed, suggesting an impact on syntrophic interactions. Sampling and technical difficulties were encountered around 80 days post-inoculation and measurements were excluded. However, methane generating proteins were observed 80 days post-inoculation, suggesting CH_4_ production upon return to the original impeller speed.
(TIF)

S2 Fig Measurement of fixed and volatile solids. Solids were highly variable during the period of transition to an established community.
(TIF)

S3 Fig Bray-Curtis similarity (1—dissimilarity) between samples. Based on the VFA (top), microbial population (middle), and LC-MS feature measurements (bottom) showing redundant patterns across the different data types. Each pattern, or block, is explained by different events occurring within the bioreactor including community acclimation, established community function, and community response to perturbation.
(TIF)
