# Supplementary figures and images for: Identification of metabolite and protein explanatory variables governing microbiome establishment and re-establishment within a cellulose-degrading anaerobic bioreactor
Source: PLoS One. 2018 Oct 5;13(10):e0204831. doi: 10.1371/journal.pone.0204831 (PMC6173382; doi:10.1371/journal.pone.0204831)

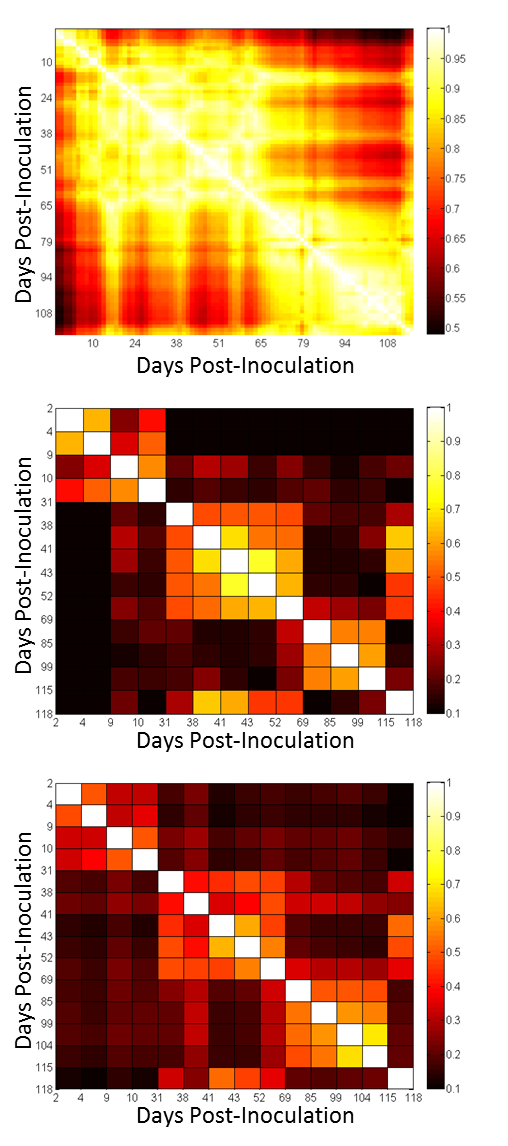

Supplement: S3 Fig — Based on the VFA (top), microbial population (middle), and LC-MS feature measurements (bottom) showing redundant patterns across the different data types. Each pattern, or block, is explained by different events occurring within the bioreactor including community acclimation, established community function, and community response to perturbation. (TIF) [file pone.0204831.s008.tif]
